# Molecular Epidemiology of Methicillin-Resistant and Methicillin-Susceptible *Staphylococcus aureus* in the Ovine Dairy Chain and in Farm-Related Humans

**DOI:** 10.3390/toxins9050161

**Published:** 2017-05-16

**Authors:** Guerrino Macori, Giuseppina Giacinti, Alberto Bellio, Silvia Gallina, Daniela Manila Bianchi, Daniele Sagrafoli, Nicla Marri, Gilberto Giangolini, Simonetta Amatiste, Lucia Decastelli

**Affiliations:** 1 National Reference Laboratory for Coagulase-Positive Staphylococci including *Staphylococcus aureus*, Istituto Zooprofilattico Sperimentale del Piemonte, Liguria e Valle d’Aosta, Via Bologna 148, 10154 Torino, Italy; alberto.bellio@izsto.it (A.B.); silvia.gallina@izsto.it (S.G.); manila.bianchi@izsto.it (D.M.B.); lucia.decastelli@izsto.it (L.D.); 2 Centro di Referenza Nazionale Della Qualità Del Latte E Dei Derivati Ovini E Caprini, Istituto Zooprofilattico Sperimentale delle Regioni Lazio e Toscana , Via Appia Nuova, 1411, 00178 Roma, Italy; giuseppina.giacinti@izslt.it (G.G.); daniele.sagrafoli@izslt.it (D.S.); nicla.marri@izslt.it (N.M.); gilberto.giangolini@izslt.it (G.G.); simonetta.amatiste@izslt.it (S.A.)

**Keywords:** *Staphylococcus aureus*, PFGE, Panton-Valentine leukocidin, MRSA, typing

## Abstract

*Staphylococcus aureus* is a major cause of clinical infections in humans and its enterotoxins cause foodborne disease. In the present study, we tested a total of 51 isolates of *S. aureus* from small-ruminant dairy farms with artisan dairy facilities, all located in Latium, Italy. The farms have a known history of a high prevalence of methicillin-resistant *S. aureus* (MRSA). Most of the MRSA isolates (27 of 51) belonged to *spa*-type t127 (43.1%), followed by t2678 (3.9%), t044 (2%), t1166 (2%), and t1773 (2%). PFGE performed on *mecA* positive strains identified one cluster (≥ 80% of similarity), comprising 22 MRSA. Nine of twenty-two MRSA isolates were assigned human host origin, and 13 isolates did not belong to a specific host. During the characterization study, one strain isolated from bulk tank milk samples harbored the *pvl* gene; the strain was not enterotoxigenic with a non-specific host according to the biotyping scheme, highlighting the possible emerging risk of transmission of bacterial virulence factors by foods, the environment, and foodhandlers. These findings stress the importance of hygienic measures at all processing steps of the food production chain and underline that monitoring for the presence of MRSA throughout the food chain is essential for public health.

## 1. Introduction

*S. aureus* is one of the most important etiological agents of intramammary infections in small ruminants [1]. In sheep it is responsible for clinical mastitis in 5–11% of cases and for sub-clinical mastitis in 0.22–2.06% of cases [2,3]. Its presence is a major economic and public health concern in the milk and dairy product sector [4]. *S. aureus* contamination of dairy products can occur anywhere in the food chain, while intra-farm spread mainly occurs through milking procedures [5], where infected mammary glands are the main reservoir [6].

Previous studies have examined a limited number of strains isolated from sheep and goat livestock that showed a host specificity and close relatedness [3,7]. Despite host specificity, *S. aureus* can switch and adapt to different host species, leading to host restriction [8].

*S. aureus* virulence isolates may harbor several staphylococcal enterotoxin genes [9], including genetic variants related to specific hosts [10]. In addition, *S. aureus* strains may also harbor pore-forming toxins involved in the pathophysiology of skin infections, such as Panton-Valentine Leukocidin (PVL) [11], and antibiotic-resistance genes, including methicillin resistance mediated by the *mecA* gene chromosomally located in the staphylococcal cassette chromosome *mec* (SCC*mec*) [12].

Recent reports of an increasing number of MRSA isolates in pets and livestock have demonstrated that the livestock-associated MRSA (LA-MRSA), belonging to the clonal complex CC398, is the most frequently isolated [13,14]. In Italy, the community-acquired MRSA (CA-MRSA) ST(CC) 1 *spa*-type t127 is reported among the main lineages of MRSA isolated in livestock productions [15,16,17,18,19,20,21]. CA-MRSA clones often harbor the PVL virulence genes, more than CA-MSSA, suggesting that methicillin resistance has contributed to the success of *pvl*-positive *S. aureus* strains [22]. Furthermore, exposure to MRSA from livestock is a route for horizontal transmission between animals and people working in contact with livestock [21,23,24,25,26], and for its possible introduction in the community through the food chain [27].

Although *S. aureus* is frequently isolated in ovine milk and dairy products [16,28,29], the occurrence of MRSA in the ovine dairy chain appears to be low [17,18,29,30,31]. The aim of this study was to investigate the genetic diversity, as determined by pulsed-field gel electrophoresis (PFGE) and *spa*-types, of MRSA and MSSA isolated from samples of udder milk, raw milk, cheeses, nasal swabs of workers, and skin swabs of ewes taken from dairy sheep farms with a history of MRSA in bulk tank milk, as documented in a previous survey. Additionally, genes encoding for PVL and staphylococcal enterotoxins (SEs) were detected.

## 2. Results

Twenty-seven of the 51 isolates of *S. aureus* (52.9%) showed resistance to methicillin (MRSA), while 24 (47.1%) were susceptible (MSSA). Twenty-seven strains harbored the *mecA* gene, confirming methicillin resistance, while no isolates harbored the *mecC* gene (*mecA_LGA251_*). The 11 genes coding for SEs were investigated for all 51 strains, and 41 isolates (80.4%) tested positive for at least one SE gene. The combination of the results generated six different SE profiles, including the isolates that tested negative for all SE genes (Table 1). The *seh* gene was the most frequent profile (33.3%), followed by the negative profile (19.7%), *sec* (17.6%), and *seg* (15.7%) genes. Seven strains (13.7%) harbored both *seg-sei* genes carried together on the enterotoxin gene cluster (egc) [32], including two profiles: *seg*-*sei* detected in five isolates (9.8%) and *sec-seg-sei* in two isolates (3.9%). The genes *sea*, *seb*, *sed*, *see*, *se*l*j*, *se*l*p*, *ser* were not detected, and two of the negative isolates for SE genes tested positive for the *mecA* gene.

Six farms participated in this study, and 831 individual milk samples were obtained from sheep. Among 14 samples positive for *S. aureus*, 8 indicated MRSA (Supplemental Table S1). The number of workers is reported in Table 2; for each worker (14), nasal, oral and skin swabs were sampled. Among the 12 *S. aureus* isolated, 5 indicated MRSA.

A total of 51 isolates were typed by *spa*-typing, generating 12 profiles. Among the *mecA* positive isolates (27 of 51), 22 had *spa*-type t127 (43.1%), two had t2678 (3.9%), and one (2.0%) had the *spa*-types t044, t1166, and t1773. Among the 24 isolates that tested negative for *mecA* and *mecC* genes, four were *spa*-type *t*528 (7.8%), three were spa-types st127, t1166, and t1773 (5.9% each), two were *spa*-types t050, t709, t902, and t3583 (3.9% each), and one was *spa*-types t084, t998, and t2678 (2.0% each).

The exotoxin PVL gene was detected in one MRSA strain isolated from bulk tank milk that tested negative for SE genes and not-specific host type 2 (NSH2) in biotyping (Table 3).

**Figure 1:**
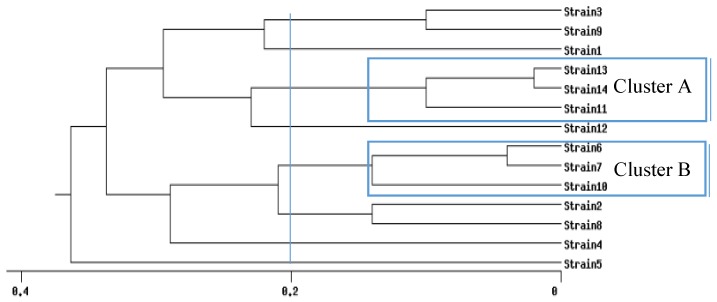
Determination of the clonal profile of *Staphylococcus aureus* strain profiles highlighted by PFGE. Dendrogram generated using the Dice/Unweighted pair group method using arithmetic averages (UPGMA) analysis (BioNumerics, Applied Maths, Version 7.1, Sint-Martens-Latem, Belgium, 2016).

Pulsed-field gel electrophoresis (PFGE) (http://insilico.ehu.es/dice_upgma/index.php) identified 13 *S. aureus* pulse types. According to Mello and colleagues [33], analysis of the PFGE profiles highlighted two *S. aureus* clusters that simultaneously included ≥ 3 isolates with similarity ≥ 80% (Figure 1).

Cluster A consisted of three strains all harboring the gene *sec* coding for SE type C. In terms of host specificity, all strains were related to an ovine origin. This cluster comprised nine isolates from farm 3 (Table 4), and all strains were MSSA.

Cluster B comprised 22 MRSA isolated from different farms (1, 2, 3, and 4). In terms of host specificity, nine isolates were assigned to human host origin and 13 isolates did not belong to a specific host (NSH3, NSH6, and NSH4) (Table 5).

## 3. Discussion

In this study, a PVL positive strain (*lukS-lukF+*) was highlighted during a study for the screening of MRSA in six ovine farms that tested positive in a previous study. PVL is one of the most important virulence factors produced by *S. aureus*, contributing to the pathogenicity of this microorganism. This toxin is associated with different diseases in humans, such as pneumonia and necrotizing dermatitis [34]. The Panton-Valentin leukocydin gene (*pvl*) gene has also been identified in *S. aureus* strains isolated from cases of mastitis in animals [35,36,37]. In Europe, the most common clone is the European ST80 [38]; however, this is the first report of *S. aureus* isolated from ovine raw milk. The strain tested positive for the *mecA* gene and negative for the enterotoxin genes tested (SEs profile number 2); it belonged to *spa*-type t044 and showed a non-host specificity (NSH2) in biotyping. The PFGE profile of the strain and the phylogenetic study showed a high genomic distance (strain 5, Figure 1) from the population studied, and it belonged to ST80, which, according to the *spa*-ridom server, is widely disseminated in Europe.

This study revealed the presence of multiple MRSA strains among animals at the farms with possible human contamination. In fact, 11 of the *mecA* positive strains confirmed by PCR (27 strains in total) indicated a human host origin in biotyping. The five strains isolated from the operators (nasal swabs, oral swab, and skin swab) from two farms (3 and 4) were considered the common infectious agent for the individual milk samples (3 isolates), bulk tank milk (2 samples), and ovine swab skin (1 isolate). Although the isolates showed the same *spa*-type (t127), they highlighted a different PFGE profile using SmaI between the strains isolated from two operators (*spa*-type t127 and PFGE profile 6) and the strains isolated from the individual milk samples of two animals (t127 and PFGE profile 7), even though the strains are genetically correlated and belong to the same cluster (Figure 1). Surprisingly, the strain isolated from the operator from farm 4 (not correlated with farm 3) had the same genetic profiles (t127 and PFGE profile 7) as the two strains isolated from the individual milk samples of animals from farm 3. Furthermore, when all the isolates with different biotyping results but belonging to cluster B were compared, the MRSA strains were present at all four farms (1, 2, 3, and 4) and were isolated from different samples, including the cheeses ready for commercialization (farms 1 and 4). Interestingly, four isolates (ID 2, ID8, ID11, and ID15) had *spa*-type t1773 and one carried the *mecA* gene (negative for the *mecA_LGA251_*), contrary to previous studies [39,40,41,42] that reported spa-type t1773 isolates positive for *mecA_LGA251_*. In those studies, the strains were isolated from cats (skin) [41,42] and humans [39,40]. In the present study, the ID2 MRSA isolate (*mecA* positive) was isolated from bulk tank milk (Table S1) and had a human origin according to the biotyping scheme. The other three strains were isolated from an individual milk sample (ID8), mammary pustules (ID11), and skin swab (ID15) from the same animal and had ovine origin according to the biotyping scheme.

*S. aureus* usually shows limited host specificity, though transfer between different host species may occur [43]. The transmission of milk-associated *S. aureus* strains between cows and humans was suggested by Lee [44] who found MRSA in milk samples with comparable antibiotypes as those in humans, but the transfer to humans was not demonstrated as, for example, the transmission from ovines to humans and vice versa. While molecular epidemiological typing did not reveal the spread of MRSA from animals to humans, it did reveal the spread of MRSA strains among ovines on two farms.

It should be emphasized that the study of *S. aureus* populations isolated from small ruminants holds importance since information is scarce. Recent detection of new *spa* types [45] and the *pvl* strain in this study suggests that the characterization of *S. aureus* isolates from ovine and caprine livestock should be encouraged.

Detection of MRSA in sheep herds is unexpected, given that the farm management practices differ from those for cattle. In fact, in small ruminant herds, breeders often prefer culling to antimicrobial treatments, resulting in a less selective pressure on *S. aureus* populations.

The presence of MRSA in farm animals is a serious health hazard for the community and could pose a difficult challenge for treating infections in humans. In Italy, Alba and colleagues observed that the MRSA strains isolated from cattle have a very high genetic relatedness with human isolates [20]. Therefore, it might be important to genetically correlate strains isolated from sheep farms to human isolates in the same area, investigating genomic features, including the arginine catabolic mobile element (ACME), which was identified in a specific clone of MRSA, called USA300 [46]. Lastly, the investigation of these genes can be useful for control the spread of possible new MRSA clones, studying their genetic evolution and the horizontal acquisition of novel genetic elements of strains isolated in different host species.

## 4. Conclusions

Characterization of SE genes in strains isolated in dairy farms is helpful for the indirect monitoring of eventual enterotoxigenic strains present in the food chain. The enterotoxin G and H genes exhibited the highest prevalence in the MRSA isolates in this study. Since there are no methods available for the identification of these enterotoxins in foodstuffs, an underestimation of the risks for humans can be assumed [47,48,49]. In addition, the potential risk for antimicrobial resistance in the food chain has to be taken into account [47,48,49]. On the other hand, the reported enterotoxin type C gene found in the MSSA, but not in the MRSA isolates, confirmed its diffusion in ovine isolates, as previously reported [28,50,51]. The description of an isolate harboring the gene for *pvl* poses a risk for the dissemination of this toxin in sheep herds. There are no directives for mandatory screening of this virulence factor in food sources. The results of this study highlight the need for more detailed investigations to improve consumer protection and public health safety.

## 5. Materials and Methods

### 5.1. Sample Collection 

A total of 51 *S. aureus* strains were collected from six dairy sheep farms, including two farms with artisan dairy facilities, all located in Latium, Italy, between 2013 and 2015. The farms had a history of MRSA in bulk tank milk, as documented in a previous survey carried out between 2012 and 2015. Five farms operated a semi-extensive breeding system, two of which also had an artisan cheese factory, while the sixth farm operated an intensive system. The circulation of MRSA and of MSSA was investigated in four of the six positive farms. The flock size ranged from 83 to 556 lactating ewes. The ewes were milked twice daily using a milking machine. The workers wore dedicated coveralls and boots, but not gloves, when handling the animals. Teat-washing before milking and treatment with antibiotics at dry-off were not performed. The dairies made raw milk cheeses, and the farm owners were usually involved in milking and cheese making.

Individual milk samples were collected from all lactating ewes in all four herds. At the end of milking, bulk tank milk was collected. Nasal and skin samples were taken using cotton-tipped swabs (Amies Agar Gel with Charcoal, Laboindustria s.p.a., Italy) and Sodibox^®^ wipes (Sodibox, Névez, France), respectively, from the farm owners and workers. None of the subjects reported recent hospitalization or the presence of a healthcare worker in the household. Two raw cheese samples were taken from each dairy plant. All samples were immediately transported to the laboratory in ice-cooled containers and analyzed within 24 h.

Overall, the isolates were obtained from udder milk (*n* = 17), mammary pustules (*n* = 2), bulk ta*n*k milk (*n* = 11), *n*asal swabs of workers (*n* = 8), skin swabs of workers (*n* = 4), skin swabs of ewes (*n* = 4), environmental swabs (*n* = 3), and raw cheese (*n* = 2). Isolates were confirmed as *S. aureus* by modified species-specific PCR, using primers targeting the *fem*A gene (132 bp) [52] and screened for methicillin resistance using the disk diffusion method according to Clinical Laboratory Standards Institute (CLSI) criteria. All human samples were obtained voluntarily, and the farm owner consented to animal sampling (in Italy, MRSA infection in animals is not a notifiable disease). All procedures followed were in accordance with ethical standards of the relevant national and institutional committees on experimentation and with the Helsinki Declaration of 1975, as revised in 2008. Farm workers gave oral informed consent to participate in the study.

### 5.2. Typing of the Bacterial Isolates

All isolates were analyzed by phenotypic tests for growth on Baird Parker rabbit plasma fibrinogen agar (Merck, Darmstadt, Germany). DNA was extracted using the InstaGene™ Matrix (Bio-Rad, Milan, Italy). Staphylococcal enterotoxin genes (SEs) were detected using two multiplex PCR (mPCR) assays according to the protocols of the European Union Reference Laboratory for Coagulase-Positive Staphylococci, including *Staphylococcus aureus* (EU-RL CPS) (ANSES—Mesons Alfort, France) [53]. The first mPCR assay detects genes encoding SEs A to E and R; the second detects genes encoding SEs G to J and P. Reference strains of *S. aureus* provided by the EU-RL CPS, FRI S6 (*sea*, *seb*), FRI 137 (*seg*, *seh*, *sei*), FRI 326 (*see*), FRI 361 (*sec*, *sed*, *ser*), and HMPL 280 (*seg*, *sei*, *se*l*j*, *se*l*p*) were used as positive controls. The biotyping scheme described by Devriese [54] was utilized to assign specific ecological variants (ecovars) associated with different host species; the method includes the production of β-hemolysin, bovine plasma coagulation, staphylokinase, and crystal violet growth reaction. The possible results are: bovine, human, ovine, and six different non-specific hosts (NSH1 to NSH6) [8].

All *S. aureus* isolates were analyzed for the presence of *mecA*, the new *mecA* homologue gene (*mecC*, formerly named *mecA_LGA251_*), and Panton-Valentine Leukocidin (PVL) (*lukS*-PV/*lukF*-PV) genes by PCR, as described previously [39]. PCR assays were performed on a GeneAmp System 9700 thermal cycler (Applied Biosystems, Foster City, CA, USA). All isolates were typed by DNA macrorestriction digestion analysis using a SmaI restriction enzyme and pulsed-field gel electrophoresis (PFGE) according to the EU-RL CPS protocol [55] modified as follows. Briefly, electrophoretic separation of DNA fragments was performed on 1% agarose gel; electrophoresis was performed using the CHEF MAPPER System (Bio-Rad Laboratories, Hercules, CA, USA); the gel was stained with GelRed (Biotium, Hayward, CA, USA) 1X solution in ultrapure water for 30 min. The restriction profiles were analyzed with BioNumerics software ver. 7.1 (Applied Maths, Sint-Martens-Latem, Belgium) using the Dice correlation coefficient and then visualized as a dendrogram by the unweighted pair-group method using arithmetic averages (UPGMA), with 1% tolerance and 1% optimization settings. Similarity cutoff values of 90%, 75%, and 70% were used to define a cluster. The polymorphic X region of the protein A gene (*spa*) was sequenced to assign the *spa*-type [56] using BioNumerics software ver. 7.1.

## Figures and Tables

**Table 1:** Results of the enterotoxin genotyping scheme.

Staphylococcoal Enterotoxin (SE) Profile	Staphylococcal Enterotoxin Gene(s)	No. of Isolates (%)
1	*seh*	17 (33.3)
2	Negative	10 (19.7)
3	*sec*	9 (17.6)
4	*seg*	8 (15.7)
5	*seg-sei*	5 (9.8)
6	*sec-seg-sei*	2 (3.9)

*seh*: staphylococcal enterotoxin type H gene; *sec*: staphylococcal enterotoxin type C gene; *seg*: staphylococcal enterotoxin type G gene; *sei*: staphylococcal enterotoxin type I gene.

**Table 2:** Farm owners and farm workers tested.

	Farm Number	Farm Workers/Owners Tested	*S. aureus*	MRSA
	1	5	2	1
	2	2	4	2
	3	4	3	1
	4	1	3	1
	5	1	0	0
	6	1	0	0
Total	6	14	12	5

MRSA: methicillin-resistant *S. aureus*.

**Table 3:** Results of the typing scheme.

ID	*pvl*	*mecA*	SEsP	*spa*-type	SmaI	Biotype	ID	*pvl*	*mecA*	SEsP	*spa*-type	SmaI	Biotype
**1**	−	+	4	t1166	6	NSH4	**27**	+	+	2	t044	5	NSH2
**2**	−	+	4	t1773	7	Human	**28**	−	+	1	t127	6	NSH3
**3**	−	+	4	t127	6	NSH3	**29**	−	+	1	t127	6	NSH6
**4**	−	+	4	t127	6	NSH3	**30**	−	+	1	t127	7	Human
**5**	−	+	4	t127	7	Human	**31**	−	−	5	t709	2	Human
**6**	−	+	4	t127	7	Human	**32**	−	−	5	t709	2	Human
**7**	−	+	4	t2678	7	Human	**33**	−	−	6	t050	8	Human
**8**	−	−	2	t1773	12	NB	**34**	−	+	1	t127	4	Human
**9**	−	−	3	t3583	13	Ovine	**35**	−	−	6	t050	8	Human
**10**	−	−	3	t3583	13	Ovine	**36**	−	+	1	t127	6	NSH3
**11**	−	−	3	t1773	13	Ovine	**37**	−	+	1	t127	6	NSH3
**12**	−	−	3	t1166	13	Ovine	**38**	−	+	1	t127	6	NSH3
**13**	−	+	2	t2678	7	Human	**39**	−	+	1	t127	6	NSH3
**14**	−	−	2	t127	12	Ovine	**40**	−	+	1	t127	6	NSH3
**15**	−	−	3	t1773	13	Ovine	**41**	−	+	1	t127	6	NSH3
**16**	−	−	3	t2678	14	Ovine	**42**	−	+	1	t127	9	Human
**17**	−	+	4	t127	6	NSH3	**43**	−	−	2	t084	10	NSH6
**18**	−	−	2	t528	1	Ovine	**44**	−	+	1	t127	6	Human
**19**	−	−	2	t528	1	Ovine	**45**	−	−	3	t998	11	Ovine
**20**	−	−	5	t902	2	Human	**46**	−	−	3	t1166	11	Ovine
**21**	−	−	2	t127	3	NSH2	**47**	−	−	3	t1166	14	Ovine
**22**	−	+	1	t127	4	NSH3	**48**	−	+	1	t127	6	Human
**23**	−	+	1	t127	4	NSH3	**49**	−	−	5	t902	2	Human
**24**	−	−	2	t528	3	NSH3	**50**	−	+	1	t127	6	Human
**25**	−	−	2	t528	3	NSH2	**51**	−	−	5	t127	2	Human
**26**	−	+	1	t127	4	NSH3							

ID: Identification number of the isolate; *pvl*: Panton-Valentine Leukocidin; *mecA*: methicillin resistance gene; SEsP: staphylococcal enterotoxin profile resulting from the combination of the 11 SE genes detected; *spa*-type: staphylococcal protein A genotype; smaI: PFGE enzyme smaI result; NB: combination of the results did not belong to any biotype described in the literature.

**Table 4:** Strains belonging to cluster A.

	ID	Farm	Type of Sample	*pvl*	*SEs* Gene	Biotyping	*spa*-type	smaI
	45	3	Mastitis udder swab	−	*sec*	Ovine	t998	11
	46	3	Udder	−	*sec*	Ovine	t1166	11
	9	3	Individual milk	−	*sec*	Ovine	t3583	13
	10	3	Individual milk	−	*sec*	Ovine	t3583	13
	11	3	Mammary pustules	−	*sec*	Ovine	t1773	13
	12	3	Mammary pustules	−	*sec*	Ovine	t1166	13
	15	3	Swab skin (sheep)	−	*sec*	Ovine	t1773	13
	47	3	Swab skin (sheep)	−	*sec*	Ovine	t1166	14
	16	3	Swab skin (sheep)	−	*sec*	Ovine	t2678	14
Total	9	1			*sec (*100.0*%)*			

ID: Identification number of the isolate; Farm: number of the farm where the strain was isolated; *pvl*: Panton-Valentine Leukocidin gene; *SEs*: staphylococcal enterotoxin gene genotype; *spa*: staphylococcal protein A typing result; smaI: PFGE enzyme smaI result.

**Table 5:** Strains belonging to cluster B.

	ID	Farm	Type of Sample	*pvl*	*SEs* Gene	Biotyping	*spa*-type	smaI
	1	4	Bulk milk	−	*seg*	NSH4	t1166	6
	2	3	Bulk milk	−	*seg*	Human	t1773	7
	3	1	Bulk milk	−	*seg*	NSH3	t127	6
	4	1	Cheese	−	*seg*	NSH3	t127	6
	5	3	Individual milk	−	*seg*	Human	t127	7
	6	3	Individual milk	−	*seg*	Human	t127	7
	7	3	Individual milk	−	*seg*	Human	t2678	7
	13	3	Swab skin (ewe)	-	neg	Human	t2678	7
	17	4	Cheese	−	*seg*	NSH3	t127	6
	28	4	Individual milk	−	*seh*	NSH3	t127	6
	29	4	Individual milk	−	*seh*	NSH6	t127	6
	30	4	Swab skin (human)	−	*seh*	Human	t127	7
	36	2	Bulk milk	−	*seh*	NSH3	t127	6
	37	1	Bulk milk	−	*seh*	NSH3	t127	6
	38	1	Bulk milk	−	*seh*	NSH3	t127	6
	39	1	Bulk milk	−	*seh*	NSH3	t127	6
	40	1	Udder half	−	*seh*	NSH3	t127	6
	41	1	Environmental swab	−	*seh*	NSH3	t127	6
	43	1	Nasal swab	−	neg	NSH6	t084	10
	44	1	Bulk milk	−	*seh*	Human	t127	6
	48	3	Nasal swab	−	*seh*	Human	t127	6
	50	3	Nasal swab	−	*seh*	Human	t127	6
Total	22	4						

ID: Identification number of the isolate; Farm: number of the farm where the strain was isolated; *pvl*: Panton-Valentine Leukocidin; *SEs*: staphylococcal enterotoxin gene genotype; *spa*-type: staphylococcal protein A typing result; smaI: PFGE enzyme smaI result.

## Supplementary Materials

The following are available online at www.mdpi.com/2072-6651/9/5/161/s1, Table S1: Type of sample and number of the farm where the strain was isolated.
