# Supplementary material for: Molecular Epidemiology of Methicillin-Resistant and Methicillin-Susceptible Staphylococcus aureus in the Ovine Dairy Chain and in Farm-Related Humans
Source: Toxins (Basel). 2017 May 16;9(5):161. doi: 10.3390/toxins9050161 (PMC5450709; doi:10.3390/toxins9050161)
Supplement: Supplementary file 1 [file toxins-09-00161-s001.docx]

Supplementary Materials: Molecular Epidemiology of Methicillin-Resistant and Methicillin-Susceptible *Staphylococcus aureus* in the Ovine Dairy Chain and in Farm-Related Humans

**Guerrino Macori, Giuseppina Giacinti, Alberto Bellio, Silvia Gallina, Daniela Manila Bianchi, Daniele Sagrafoli, Nicla Marri, Gilberto Giangolini, Simonetta Amatiste and Lucia Decastelli**

**Table S1.** Type of sample and number of the farm where the strain was isolated.

| **ID** | **Type of Sample** | **Farm** |
| --- | --- | --- |
| 1 | Bulk tank milk | 3 |
| 2 | Bulk tank milk | 2 |
| 3 | Bulk tank milk | 1 |
| 4 | Cheese | 1 |
| 5 | Individual milk | 2 |
| 6 | Individual milk | 2 |
| 7 | Individual milk | 2 |
| 8 | Individual milk | 2 |
| 9 | Individual milk | 2 |
| 10 | Individual milk | 2 |
| 11 | mammary pustules | 2 |
| 12 | mammary pustules | 2 |
| 13 | Swab skin (sheep) | 2 |
| 14 | Swab skin (sheep) | 2 |
| 15 | Swab skin (sheep) | 2 |
| 16 | Swab skin (sheep) | 2 |
| 17 | Cheese | 3 |
| 18 | Individual milk | 3 |
| 19 | Individual milk | 3 |
| 20 | Nasal swab (farm worker 1) | 2 |
| 21 | Bulk tank milk | 4 |
| 22 | Individual milk | 4 |
| 23 | Individual milk | 4 |
| 24 | Individual milk | 4 |
| 25 | Individual milk | 4 |
| 26 | Bulk tank milk | 4 |
| 27 | Bulk tank milk | 5 |
| 28 | Individual milk | 3 |
| 29 | Individual milk | 3 |
| 30 | Swab skin (farm worker 1) | 3 |
| 31 | Swab skin (farm worker 2) | 3 |
| 32 | Swab skin (farm worker 3) | 3 |
| 33 | Nasal swab (farm worker 1) | 4 |
| 34 | Oral swab (farm worker 1) | 4 |
| 35 | Swab skin (farm worker 1) | 4 |
| 36 | Bulk tank milk | 6 |
| 37 | Bulk tank milk | 1 |
| 38 | Bulk tank milk | 1 |
| 39 | Bulk tank milk | 1 |
| 40 | Udder half | 1 |
| 41 | Environmental swab | 1 |
| 42 | Nasal swab (farm worker 1) | 1 |
| 43 | Nasal swab (farm worker 2) | 1 |
| 44 | Bulk tank milk | 1 |
| 45 | Mastitis udder swab | 2 |
| 46 | Udder | 2 |
| 47 | Swab skin (sheep) | 2 |
| 48 | Nasal swab (farm worker 2) | 2 |
| 49 | Nasal swab (farm worker 1) | 2 |
| 50 | Nasal swab (farm worker 2) | 2 |
| 51 | Environmental swab | 2 |
